# Can aspirin help?

**DOI:** 10.7554/eLife.35906

**Published:** 2018-03-21

**Authors:** Ashwini Kalantri, Shriprakash Kalantri

**Affiliations:** 1 Department of Community MedicineMahatma Gandhi Institute of Medical SciencesSevagramIndia; 2 Department of MedicineMahatma Gandhi Institute of Medical SciencesSevagramIndia

**Keywords:** tuberculous meningitis, aspirin, stroke, clinical trial, Mycobacterium tuberculosis, tuberculosis, Human

## Abstract

Using a combination of aspirin, anti-tuberculosis drugs and steroids may help to reduce the number of strokes and deaths in patients with tuberculous meningitis.

**Related research article** Mai NT, Dobbs N, Phu NH, Colas RA, Thao LT, Thuong NT, Nghia HD, Hanh NH, Hang NT, Heemskerk AD, Day JN, Ly L, Thu DD, Merson L, Kestelyn E, Wolbers M, Geskus R, Summers D, Chau NV, Dalli J, Thwaites GE. 2018. A randomised double blind placebo controlled phase 2 trial of adjunctive aspirin for tuberculous meningitis in HIV-uninfected adults. *eLife* **7**:e33478. doi: 10.7554/eLife.33478

Tuberculosis is a highly infectious disease that, over the millennia, has killed more people than wars or famines (World Health Organization, 2017). Roughly one third of the world's population – almost two billion people – are thought to carry the pathogen that causes the disease. It particularly affects low-income countries, and almost two million people died from the disease in such countries in 2017 (Dheda et al., 2016).

The most lethal and disabling form of tuberculosis is tuberculous meningitis, which causes inflammation in the brain (Wilkinson et al., 2017). This type of tuberculosis happens when the bacteria move to the protective covering of the brain, the meninges, and form small lesions that can rupture and so prompt the symptoms of the disease. Most untreated patients die within two months of the onset of illness (Thwaites et al., 2013).

Tuberculous meningitis (TBM) is commonly treated with a combination of steroids (to reduce the inflammation) and anti-tuberculosis drugs (to fight the bacteria). Although this combination of treatments can improve survival, the mortality rates and severe cases of disability continue to be unacceptably high (Heemskerk et al., 2016; Thwaites et al., 2004). People with TBM often suffer from complications due to fluids that accumulate in the brain cavities, and from inflamed blood vessels that can lead to blood clots, the main cause of strokes. Could drugs that prevent clots and reduce inflammation help patients with TBM?

Now, in eLife, Guy Thwaites and colleagues – including Nguyen Mai as first author – report that a common anti-inflammatory drug may hold the key to answering this question (Mai et al., 2018). The researchers – who are based in Vietnam, the UK and the Netherlands – designed a clinical trial to test the efficacy and safety of aspirin in patients with TBM.

Aspirin is a drug commonly used to avert or treat heart attacks and strokes. It prevents blood clots from forming and also reduces inflammation. Patients taking aspirin suffer fewer heart attacks, strokes and deaths than those who do not, albeit at the cost of a small number of bleeding events (Antithrombotic Trialists' (ATT) Collaboration et al., 2009).

In the experiments, 120 Vietnamese adults were randomly assigned to receive a low dose of aspirin (81 mg/day), a high dose of aspirin (1000 mg/day), or an identical tablet that did not contain any medication (called the placebo). All groups were also given anti-tuberculosis drugs and steroids for the first 60 days. Mai et al. then assessed the efficacy of aspirin by analyzing brain scans and measuring markers of thrombosis and inflammation in the fluid surrounding the brain and the spinal cord.

The results showed that aspirin reduced stroke-associated disability and death, at least for a short time. Compared to the placebo, a low dose of aspirin lowered the risk by 7%, and a high dose by 13%. However, given the small number of patients in the study, these results should be interpreted with caution. Encouragingly though, the risk of bleeding in the brain or stomach did not increase significantly in patients receiving aspirin, regardless of the dose. Thus, this trial suggests that aspirin is safe when used alongside the drugs normally used to treat TBM.

In March 1882, Robert Koch discovered the bacterium that causes tuberculosis – a turning point in our understanding of the disease (Blevins and Bronze, 2010). A century later, accurate diagnosis and potent drugs have helped to reduce the number of deaths caused by this illness. But researchers and physicians have failed to replicate this success story in TBM.

Could aspirin, used in combination with standard treatments, cut the Gordian knot to solve or even prevent the complications associated with TBM? Could it improve long-term survival and offer disability-free life to patients? Mai et al. show that aspirin might be able to achieve these goals to some extent. The trial was, however, not designed to answer all these questions. A larger phase 3 trial, which compares this new approach with the best currently available treatment, will be needed to confirm that aspirin is indeed safe and able to reduce the number of strokes and deaths in patients with TBM.
